# Substantia nigral dopamine transporter uptake in dementia with Lewy bodies

**DOI:** 10.1038/s41531-023-00534-9

**Published:** 2023-06-09

**Authors:** Young-gun Lee, Seun Jeon, Kyoungwon Baik, Sung Woo Kang, Byoung Seok Ye

**Affiliations:** 1 Department of Neurology, Yonsei University College of Medicine, Seoul, South Korea; 2 Department of Neurology, Ilsan Paik Hospital, Inje University College of Medicine, Goyang, South Korea; 3 Brain Research Institute, Yonsei University College of Medicine, Seoul, South Korea

**Keywords:** Diagnostic markers, Neurodegeneration, Parkinson's disease

## Abstract

Nigrostriatal dopaminergic degeneration is a pathological hallmark of dementia with Lewy bodies (DLB). To identify the subregional dopamine transporter (DAT) uptake patterns that improve the diagnostic accuracy of DLB, we analyzed N-(3-[^18^F] fluoropropyl)-2β-carbomethoxy-3β-(4-iodophenyl)-nortropane (FP-CIT) PET in 51 patients with DLB, in 36 patients with mild cognitive impairment with Lewy body (MCI-LB), and in 40 healthy controls (HCs). In addition to a high affinity for DAT, FP-CIT show a modest affinity to serotonin or norepinephrine transporters. Specific binding ratios (SBRs) of the nigrostriatal subregions were transformed to age-adjusted z-scores (zSBR) based on HCs. The diagnostic accuracy of subregional zSBRs were tested using receiver operating characteristic (ROC) curve analyses separately for MCI-LB and DLB versus HCs. Then, the effect of subregional zSBRs on the presence of clinical features and gray matter (GM) density were evaluated in all patients with MCI-LB or DLB as a group. ROC curve analyses showed that the diagnostic accuracy of DLB based on the zSBR of substantia nigra (area under the curve [AUC], 0.90) or those for MCI-LB (AUC, 0.87) were significantly higher than that based on the zSBR of posterior putamen for DLB (AUC, 0.72) or MCI-LB (AUC, 0.65). Lower zSBRs in nigrostriatal regions were associated with visual hallucination, severe parkinsonism, and cognitive dysfunction, while lower zSBR of substantia nigra was associated with widespread GM atrophy in DLB and MCI-LB patients. Taken together, our results suggest that evaluation of nigral DAT uptake may increase the diagnostic accuracy of DLB and MCI-LB than other striatal regions.

## Introduction

Lewy-related pathology (LRP) is the pathologic hallmark of dementia with Lewy bodies (DLB)^1^. Currently, no direct in vivo biomarker for LRP is clinically available, and the diagnosis of DLB is based on the presence of core clinical features such as rapid eye movement sleep behavior disorder (RBD), visual hallucination (VH), cognitive fluctuation (CF), and parkinsonism^2^. Among imaging biomarkers, indirect measurement of nigrostriatal dopaminergic degeneration by dopamine transporter (DAT) positron emission tomography (PET) or single-photon emission computed tomography (SPECT) showed modest diagnostic accuracy^3,4^, as an indicative biomarker in the consensus guidelines of DLB^2,5^.

Although 2β-carbomethoxy-3β-(4-iodophenyl)-N-(3-fluoropropyl) nortropane (FP-CIT) PET showed over 90% diagnostic sensitivity in Parkinson’s disease (PD), which is on the same spectrum of α-synucleinopathy^1^, variable diagnostic accuracy has been reported in the diagnosis of DLB^3,4,6^. In a study comparing FP-CIT PET patterns between DLB and PD, more diffuse and less asymmetric striatal FP-CIT uptake was reported in DLB^7^. As shown in autopsy-imaging correlation studies, patients with mild dopaminergic neuronal loss and limited α-synuclein pathology in the substantia nigra pars compacta had normal findings in FP-CIT PET^8^. Considering that LRP could spread not only following a caudo-rostral pattern but also originating from the amygdala^9^, and that limbic or cortical predominant LRPs are common in pathologic findings of patients with DLB^2,10^, a heterogenous FP-CIT uptake pattern could be expected in patients with DLB. Research criteria for the diagnosis of pre-dementia stages of DLB, termed as mild cognitive impairment with Lewy bodies (MCI-LB), has been proposed for the early diagnosis and intervention of DLB^5^. Assessment of clinically probable MCI-LB along with DLB would be important to understand the temporal involvement of clinical symptoms and evolution of imaging biomarkers, as the clinical manifestations could develop before the onset of dementia. In addition, evidence suggests the higher involvement of nigrostriatal degeneration in MCI-LB, although lower sensitivity of DAT scans, has been reported^11,12^.

DAT is exclusively expressed in dopaminergic neurons. It is transported from the soma of dopaminergic neurons to be enriched in the terminal axon in the striatum^13,14^. However, it is also transported to the dendrites to autoinhibit and control the local release of dopamine^14,15^. We hypothesized that decreased FP-CIT uptake in the substantia nigra could reflect early dysfunction of dopaminergic neurons and could be an optimal region of interest (ROI) for the clinical diagnosis of DLB. In this study, we conducted receiver operating characteristic (ROC) curve analyses for the diagnostic accuracy of subregional FP-CIT-specific binding ratio (SBR) in the nigrostriatal areas of the MCI-LB or DLB versus healthy controls (HCs). Furthermore, we evaluated the effect of nigrostriatal FP-CIT SBR on the clinical symptoms, severity of parkinsonism, cognition, and gray matter (GM) density in the spectrum of LB disease, encompassing both MCI and dementia stage, as a group.

## Results

### Demographic and clinical characteristics

The demographic and clinical characteristics of the participants are shown in Table 1. At the time of FP-CIT PET, the HCs were younger and were educated to a higher level than patients with MCI-LB (*n* = 36) and DLB (*n* = 51). No significant differences were noted in the proportion of males and females between the subgroups. Durations from the diagnosis of the disease to time when FP-CIT PET was conducted was not different between MCI-LB and DLB. All core clinical symptoms of DLB, including RBD, CF, VH and modest parkinsonism, defined as Unified Parkinson’s Disease Rating Scale part III (UPDRS-III) ≥ 20, were observed only in patients with MCI-LB or DLB. The UPDRS-III was higher in patients with MCI-LB or DLB than HCs. The sum of boxes of Clinical Dementia Rating® (CDR-SOB) were highest and the Korean version of the mini-mental status examination (K-MMSE) score was lowest in patients with DLB, followed by MCI-LB and then HCs. The proportion of prescriptions for selective serotonin reuptake inhibitors was significantly higher in patients with MCI-LB and DLB than HCs, while prescriptions for antipsychotics was significantly higher in patients with DLB than HCs. The MCI-LB and DLB groups had lower mean SBRs in all five regions than the HC group, and substantia nigra (SN)-SBR was lower in the DLB group than the MCI-LB group (Supplementary Fig. 1). Comparison of age-adjusted z-score of SBRs (zSBRs) based on HC subgroup was similar to SBRs, while posterior putamen (PP)-zSBR was comparable between MCI-LB and HCs.

**Table 1:** Demographic and clinical characteristics of the participants.

	HC (*N* = 40)	MCI-LB (*N* = 36)	DLB (*N* = 51)	*P*
Age at FP-CIT PET, years	65.2 (± 9.3)^a, b^	76.4 (± 7.5)^a^	77.6 (± 6.1)^b^	<0.001
Duration since diagnosis, years		0.4 (± 0.5)	0.2 (± 0.3)	0.056
Follow-up duration, years		3.0 (± 1.8)	2.8 (± 1.6)	0.610
Female, no. (%)	20 (50.0)	20 (55.6)	28 (54.9)	0.861
Education, years	14.2 (± 3.8)^a, b^	8.1 (± 5.4)^a^	9.1 (± 5.7)^b^	<0.001
RBD, no (%)	0 (0.0)^a, b^	28 (77.8)^a^	29 (56.9)^b^	<0.001
Cognitive fluctuation, no (%)	0 (0.0)^a, b^	12 (33.3)^a^	27 (52.9)^b^	<0.001
Visual hallucination, no (%)	0 (0.0)^a, b^	7 (19.4)^a, c^	26 (51.0)^b, c^	<0.001
Parkinsonism, no (%)	0 (0.0)^a, b^	36 (100.0)^a^	48 (94.1)^b^	<0.001
UPDRS-III	1.4 (± 3.2)^a, b^	30.2 (± 7.9)^a^	32.7 (± 9.6)^b^	<0.001
K-MMSE	29.0 (± 1.1)^a, b^	24.0 (± 4.1)^a, c^	19.8 (± 3.9)^b, c^	<0.001
CDR-SOB	0.1 (± 0.2)^a, b^	1.6 (± 1.0)^a, c^	6.1 (± 2.6)^b, c^	<0.001
Medication, no (%)				
SSRI	0 (0.0)^a, b^	7 (19.4)^a^	14 (27.5)^b^	0.002
Antipsychotics	0 (0.0)^b^	4 (11.1)	8 (15.7)^b^	0.037
Levodopa	0 (0.0)	3 (8.3)	7 (13.7)	0.054
AP-SBR	3.5 (± 0.6)^a, b^	2.8 (± 0.8)^a^	2.4 (± 1.0)^b^	<0.001
PP-SBR	3.0 (± 0.5)^a, b^	2.4 (± 0.8)^a^	2.0 (± 1.0)^b^	<0.001
AC-SBR	3.1 (± 0.6)^a, b^	2.2 (± 0.7)^a^	1.9 (± 0.8)^b^	<0.001
PC-SBR	2.0 (± 0.5)^a, b^	1.3 (± 0.5)^a^	1.2 (± 0.5)^b^	<0.001
SN-SBR	1.1 (± 0.2)^a, b^	0.8 (± 0.2)^a, c^	0.5 (± 0.2)^b, c^	<0.001
AP-zSBR	0.0 (± 1.0)^a, b^	−0.9 (± 1.4)^a^	−1.5 (± 1.8)^b^	<0.001
PP-zSBR	0.0 (± 1.0)^b^	−0.8 (± 1.7)	−1.5 (± 2.0)^b^	<0.001
AC-zSBR	0.0 (± 1.0)^a, b^	−1.1 (± 1.5)^a^	−1.6 (± 1.8)^b^	<0.001
PC-zSBR	0.0 (± 1.0)^a, b^	−0.8 (± 1.2)^a^	−1.0 (± 1.2)^b^	<0.001
SN-zSBR	0.0 (± 1.0)^a, b^	−1.5 (± 1.2)^a, c^	−2.2 (± 1.4)^b, c^	<0.001

*AC* anterior caudate, *AP* anterior putamen, *CDR-SOB* Clinical Dementia Rating-sum of boxes, *DLB* Dementia with Lewy bodies, *HC* healthy control, *K-MMSE* Korean version of Mini-Mental State Examination, *MCI-LB* mild cognitive impairment with Lewy body, *PC* posterior caudate, *PP* posterior putamen, *RBD* REM sleep behavior disorder, *SBR* specific binding ratio, *SN* substantia nigra, *SSRI* selective serotonin reuptake inhibitor, *UPDRS-III* Unified Parkinson’s Disease Rating Scale Part III, *zSBR* age-adjusted z-score of SBR.

Plus-minus values are the mean±SD. Data are expressed as mean (standard deviation) or numbers (%). *P* values are results from chi-square test or analysis of variance as appropriate.

Significantly different between HC and MCI-LB^a^; HC and DLB^b^; and MCI-LB and DLB^c^.

### Diagnostic accuracy of age-adjusted SBRs for DLB and MCI-LB

Univariate logistic regression analyses performed in the combined groups consisting of DLB and HC or MCI-LB and HC showed that lower zSBRs in all five regions were associated with an increased risk of DLB or MCI-LB, and the model using SN-zSBR as a predictor had the lowest Akaike’s Information Criterion (Supplementary Table 1).

**Fig. 1:**
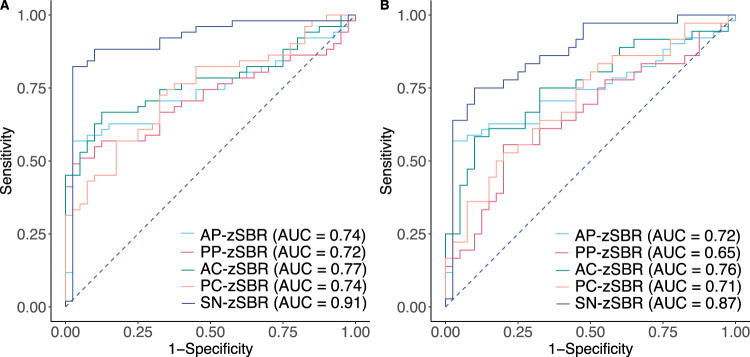
ROC curve analyses for the diagnostic accuracy of DLB and MCI-LB. The results of ROC analyses for the diagnostic accuracy of DLB (**A**) and MCI-LB (**B**) versus HCs, using nigrostriatal zSBRs as predictors. AC anterior caudate, AP anterior putamen, AUC area under the curve, DLB dementia with Lewy bodies, HC healthy control, MCI-LB mild cognitive impairment with Lewy body, PC posterior caudate, PP posterior putamen, ROC receiver operating characteristic, SN substantia nigra, zSBR age-adjusted z-score of specific binding ratio.

In the ROC curve analyses for the diagnosis of DLB versus HC, the SN-zSBR had the highest accuracy (area under the curve [AUC], 0.91; sensitivity, 82.4%; specificity, 97.5%), which was higher than that based on posterior putamen (PP)-zSBR (AUC, 0.72; sensitivity, 49.0%; specificity, 97.5%) (Fig. 1A and Supplementary Table 2). In ROC curve analyses for the diagnosis of MCI-LB versus HC, the diagnostic accuracy of SN-zSBR was lower than that for DLB (Fig. 1B and Supplementary Table 2). When mean striatal (caudate and putamen) zSBR estimates were used as predictors, diagnostic accuracy was similar to each subregional striatal zSBR, but lower than SN-zSBR (Supplementary Table 3). To evaluate whether calculation of zSBRs by combining bilateral ROIs influenced the diagnostic accuracy, we further conducted ROC curve analyses using zSBR of the more affected side, which revealed similar diagnostic accuracy (Supplementary Fig. 2).

### Conditional probability analysis of abnormal age-adjusted DAT uptake for DLB and MCI-LB

**Fig. 2:**
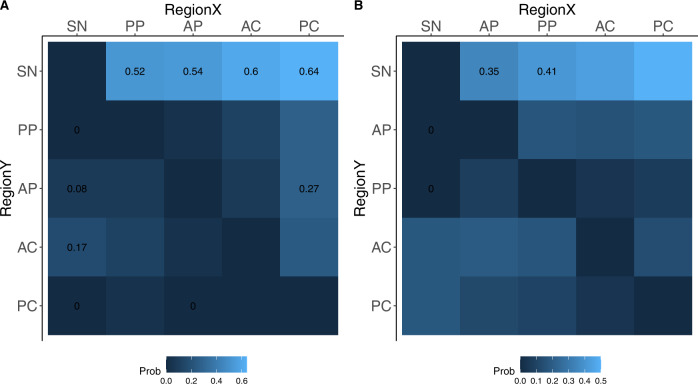
Conditional probability analysis for the sequential order of abnormal DAT uptake in nigrostriatal subregions. Pairwise matrix of the probability of abnormal DAT uptake in region Y given normal DAT uptake in region X, denoted as ${\rm{P}}\left(Y+|X-\right)$, in patients with DLB (**A**) and MCI-LB (**B**). Conditional probability with statistical significance at the <0.01 level using exact McNemar’s test, is calculated. AC anterior caudate, AP anterior putamen, DAT dopamine transporter, DLB dementia with Lewy bodies, MCI-LB mild cognitive impairment of Lewy body, PC posterior caudate, PP posterior putamen, SN substantia nigra.

The conditional probability analysis showed the possible sequential order of abnormal regional zSBRs (< −1.5; Fig. 2). In DLB, abnormal SN-zSBRs were observed when all striatal zSBRs remained normal, and abnormal anterior putamen (AP)-zSBR preceded abnormal posterior caudate (PC)-zSBR (Fig. 2A). In MCI-LB, abnormal SN-zSBRs were observed when AP- and PP-zSBR remained normal (Fig. 2B).

### Effect of age-adjusted SBRs on the clinical features and cognitive scores in the overall patient group

To investigate the effects of zSBRs on the spectrum of both MCI-LB and DLB, logistic regression analyses performed in the overall patient group showed that lower zSBRs in all nigrostriatal subregions except posterior caudate were associated with an increased risk of VH (Table 2). To rule out the possibility of adverse effects of antipsychotics for inducing VH, we further included the medication history of antipsychotics for sensitivity analysis in the logistic regression analyses for VH (Supplementary Table 4). The effects of zSBRs on VH were maintained even after consideration for antipsychotics. Although zSBRs in any region were not associated with the modest parkinsonism (UPDRS-III > 20), zSBRs in all regions were associated with higher UPDRS-III (Table 3 and Supplementary Fig. 3).

**Table 2:** Univariable analyses for the effects of nigrostriatal FP-CIT zSBRs on core clinical symptoms for the overall patient group.

	RBD		CF		VH		Parkinsonism^a^	
	OR (95% CI)	*P*	OR (95% CI)	*P*	OR (95% CI)	*P*	β (95% CI)	*P*
AP-zSBR	0.98 (0.71–1.34)	0.879	0.90 (0.67–1.21)	0.500	0.63 (0.44–0.87)	**0.007**	0.96 (0.40–2.19)	0.921
PP-zSBR	0.99 (0.75 – 1.29)	0.918	0.89 (0.68–1.14)	0.359	0.69 (0.51–0.91)	**0.011**	0.79 (0.33–1.60)	0.545
AC-zSBR	1.02 (0.74–1.43)	0.887	0.86 (0.63–1.18)	0.357	0.64 (0.44–0.90)	**0.013**	1.10 (0.45–2.84)	0.836
PC-zSBR	0.91 (0.58–1.44)	0.694	0.91 (0.59–1.38)	0.647	0.62 (0.38–0.98)	0.050	0.85 (0.26–3.23)	0.793
SN-zSBR	0.95 (0.65–1.38)	0.778	1.04 (0.73–1.48)	0.836	0.59 (0.38–0.87)	**0.010**	0.46 (0.11–1.29)	0.185

*AC* anterior caudate, *AP* anterior putamen, *CF* cognitive fluctuation, *CI* confidence interval, *DLB* dementia with Lewy bodies, *MCI-LB* mild cognitive impairment with Lewy body, *OR* odds ratio, *PC* posterior caudate, *PP* posterior putamen, RBD REM sleep behavior disorder, *SN* substantia nigra, *VH* visual hallucination, *zSBR* age-adjusted z-score of specific binding ratio.

^a^Unified Parkinson’s Disease Rating Scale Part III > 20

Data are results of logistic regression analyses for the presence of RBD, CF, VH, or parkinsonism using nigrostriatal zSBRs as predictors in patients with DLB and MCI-LB. Covariates included age, sex, and education. *P* values in bold are significant after multiple comparisons corrections across five predictors using the false discovery rate (FDR) method.

**Table 3:** Univariate analysis for the effects of nigrostriatal FP-CIT zSBRs on the UPDRS motor score, CDR-SOB, and K-MMSE in the overall patient group.

	UPDRS-III		CDR-SOB		K-MMSE	
	β^a^ (SE)	*P*	β^a^ (SE)	*P*	β^a^ (SE)	*P*
AP-zSBR	−0.25 (0.64)	**0.042**	−0.31 (0.22)	**0.015**	0.24 (0.31)	0.047
PP-zSBR	−0.35 (0.54)	**0.003**	−0.22 (0.20)	0.071	0.12 (0.27)	0.311
AC-zSBR	−0.28 (0.67)	**0.030**	−0.35 (0.24)	**0.009**	0.25 (0.33)	0.051
PC-zSBR	−0.34 (0.90)	**0.006**	−0.15 (0.33)	0.253	0.08 (0.46)	0.499
SN-zSBR	−0.30 (0.75)	**0.009**	−0.46 (0.25)	**<0.001**	0.33 (0.36)	**0.003**

*AC* anterior caudate, *AP* anterior putamen, *CDR-SOB* Clinical Dementia Rating-sum of boxes, *DLB* dementia with Lewy bodies, *K-MMSE* Korean version of Mini-Mental State Examination, *MCI-LB* mild cognitive impairment with Lewy body, *PC*, posterior caudate, *PP* posterior putamen, *SN* substantia nigra, *UPDRS-III* Unified Parkinson’s Disease Rating Scale Part III, *zSBR* age-adjusted z-score of specific binding ratio.

^a^Standardized regression coefficient

Data are results of general linear models for UPDRS-III, CDR-SOB, and K-MMSE scores using nigrostriatal zSBRs as predictors in patients with DLB and MCI-LB. Covariates included age, sex, and education. *P* values in bold is significant after multiple comparisons corrections across five predictors using the false discovery rate (FDR) method.

General linear models (GLMs) for general cognitive status showed that lower K-MMSE scores were associated with lower SN-zSBR, while higher CDR-SOB scores were associated with lower anterior putamen (AP)-zSBR, AC-zSBR, and SN-zSBR (Table 3 and Supplementary Fig. 3). GLMs for detailed neuropsychological tests showed that lower Korean version of the Boston naming test (K-BNT) scores were associated with lower zSBRs in all regions, while lower Rey–Osterrieth complex figure test (RCFT) copy and controlled oral word association test (COWAT) semantic scores were associated with lower AP-zSBR, AC-zSBR, and SN-zSBR (Table 4). Lower Seoul Verbal Learning Test (SVLT) delayed recall scores were associated with lower AC-zSBR and SN-zSBR, while lower Stroop color reading scores were associated with lower AP-zSBR and SN-zSBR.

**Table 4:** General linear models for the effects of nigrostriatal FP-CIT zSBRs on the cognitive scores in the overall patient group.

	Digit span backward		K-BNT		RCFT copy		SVLT delayed recall		COWAT semantic		Stroop color reading	
	β^a^ (SE)	*P*	β^a^ (SE)	*P*	β^a^ (SE)	*P*	β^a^ (SE)	*P*	β^a^ (SE)	*P*	β^a^ (SE)	*P*
AP-zSBR	0.05 (0.09)	0.686	0.38 (0.08)	**0.001**	0.31 (0.21)	0.005	0.25 (0.08)	0.032	0.29 (0.07)	**0.014**	0.30 (0.10)	**0.013**
PP-zSBR	0.03 (0.07)	0.780	0.33 (0.07)	**0.005**	0.14 (0.19)	0.206	0.18 (0.07)	0.101	0.21 (0.06)	0.066	0.23 (0.09)	0.051
AC-zSBR	0.04 (0.09)	0.764	0.49 (0.08)	**0.000**	0.30 (0.22)	0.010	0.28 (0.08)	**0.019**	0.29 (0.07)	**0.020**	0.27 (0.10)	0.034
PC-zSBR	0.08 (0.12)	0.511	0.42 (0.11)	**0.000**	0.05 (0.31)	0.635	0.23 (0.11)	0.048	0.22 (0.10)	0.069	0.19 (0.14)	0.130
SN-zSBR	0.08 (0.10)	0.515	0.27 (0.10)	**0.015**	0.31 (0.25)	0.002	0.36 (0.09)	**0.001**	0.34 (0.08)	**0.001**	0.30 (0.11)	**0.005**

*AC* anterior caudate, *AP* anterior putamen, *COWAT* controlled oral word association test, *DLB* dementia with Lewy bodies, *K-BNT* Korean version of Boston Naming Test, *MCI-LB* mild cognitive impairment with Lewy body, *PC* posterior caudate, *PP* posterior putamen, *RCFT* Rey–Osterrieth Complex Figure Test, *SN* substantia nigra, *SVLT* Seoul Verbal Learning Test, *zSBR* age-adjusted z-score of specific binding ratio.

^a^Standardized regression coefficient

Data are results of general linear models for cognitive scores using nigrostriatal zSBR as predictors in patients with DLB and MCI-LB. Covariates included age, sex, and education. *P* values in bold are significant after multiple comparisons corrections across five predictors using the false discovery rate (FDR) method.

### Effect of age-adjusted SBRs on the brain atrophy in the overall patient group

**Fig. 3:**
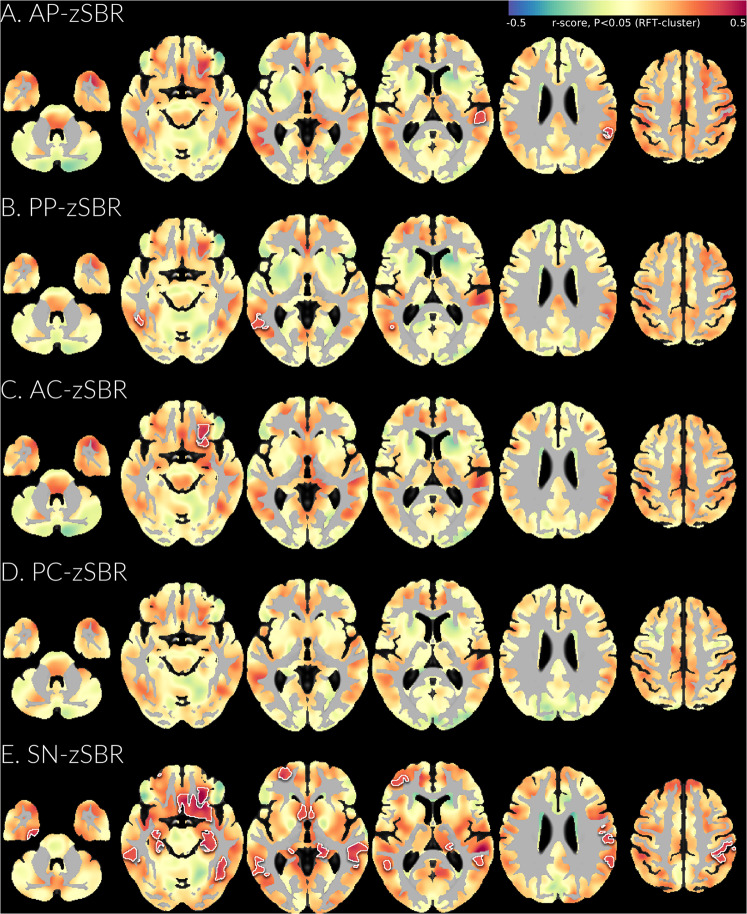
Significant gray matter changes associated with nigral zSBRs in patients with DLB and MCI-LB. The results are based on a general linear model for modulated voxel-wise gray matter using zSBR of anterior putamne (A), posterior putamen (B), anterior caudate (C), posterior caudate (D), and substantia nigra (E) as a predictor and age, sex, education, and intracranial volume as covariates. The intracranial volume is defined as the sum of gray matter, white matter, and cerebrospinal fluid volumes in the native space. The color scale indicates the effect sizes (r-scores) in statistical analysis. The areas bounded by the white line indicate brain regions that show significant correlations after correcting for multiple comparisons using random field theory cluster correction (corrected *p* < 0.05, family-wise error rate). *AC* anterior caudate, *AP* anterior putamen, *DLB* dementia with Lewy bodies, *MCI-LB* mild cognitive impairment with Lewy body, *PC* posterior caudate, *PP* posterior putamen, *SN* substantia nigra, *zSBR* age-adjusted z-score of specific binding ratio.

In the overall patient group, lower nigrostriatal SBR was associated with decreased GM density in widespread brain regions including the bilateral amygdala, inferior and lateral temporal, basal frontal, medial and lateral parietal, dorsolateral prefrontal, occipital, and perirolandic regions, although only SN-zSBR reached statistical significance after correction for multiple comparisons, while AP-zSBR, PP-zSBR, and AC-zSBR showed significance only in the limited regions (Fig. 3).

## Discussion

Here, we evaluated the diagnostic and clinical implications of regional SBRs on FP-CIT PET in patients with clinically probable DLB and MCI-LB. By using high-resolution PET and co-registration with subject-specific brain MRI, we could investigate not only subregions of striatum but also substantia nigra. Then, we investigated the effects of regional SBRs on clinical symptoms and cognition in the DLB spectrum encompassing MCI-LB and DLB. The major findings of our study were as follows. First, abnormality in SN-zSBR increased the diagnostic accuracy of DLB or MCI-LB versus HCs than striatal SBRs. Second, conditional probability showed that an abnormality in nigral zSBR preceded that in striatal zSBRs in MCI-LB and DLB. Third, regional zSBRs were associated with VH, severe parkinsonism, and cognitive dysfunction in DLB spectrum. Fourth, lower SN-zSBR was associated with widespread GM atrophy in DLB spectrum. Taken together, our results suggest that nigral DAT activity decreases early in DLB and could enhance the diagnostic accuracy of DLB and MCI-LB than striatal DAT activity.

Our first major finding was that SN-zSBR increased the diagnostic accuracy of DLB or MCI-LB versus HCs than striatal SBRs. Previous studies have shown that striatal FP-CIT PET has modest sensitivity and high specificity for the diagnosis of DLB in both clinically diagnosed and autopsy-confirmed cases^3,4,6,11,16^. However, variable diagnostic accuracy has been reported in clinically diagnosed DLB cases, particularly in MCI-LB^6,11,12^. Normal DAT imaging results have been reported in patients with neuropathologically confirmed DLB, which may be attributed to the preserved or minimally affected dopaminergic neurons in the SN^8,17–19^. In our cohort, when combined striatal zSBR estimates were used, about 41.5% and 27.8% of the patients with DLB and MCI-LB, respectively, showed normal DAT results (Supplementary Table 3). By using high-resolution PET, we showed that SN-zSBR significantly increased diagnostic accuracy compared with striatal SBRs in DLB. Due to the low resolution of SPECT, DAT uptake within the SN has been rarely described in previous studies. As DAT immunoreactivity in the midbrain is highest in the SN^20^, and DAT is expressed exclusively in the dopaminergic neurons^13,14^, loss of dopaminergic neuronal density^21^, decrease of DAT messenger RNA^22–24^, or internalization of autoregulatory DAT from the dendritic membranes^15^ as a response to dysfunctional axon terminal in the striatum may explain the decreased nigral DAT uptake in patients with DLB. In addition to DAT, FP-CIT has a modest affinity to other monoamine transporters, including serotonin transporter (SERT) and norepinephrine transporter (NET)^25,26^. Given that both serotonergic and noradrenergic neurons projects to SN^27,28^, decreased serotonergic or noradrenergic neurons projecting to the substantia nigra may contribute to lower SN-zSBR. This explanation is also consistent with previous findings that intraneuronal α-synuclein burden in locus coeruleus precedes than SN^29^, and decreased SERT or NET is associated with symptoms and severity of LBD^26,30–32^. Taken together, decreased SN-zSBR could be an early biomarker for DLB, attributed to the degeneration of dopaminergic, serotonergic, and noradrenergic neurons.

Our second major finding was that the abnormality in nigral zSBR preceded that in striatal zSBRs in DLB and MCI-LB. In addition, SN-zSBR was most prominently decreased among the regional zSBRs in the DLB group, followed by AC-zSBR (Table 1). This sequential order explains why diagnostic accuracy of PP-zSBR is lower in DLB or MCI-LB, which is the ROI in the visual inspection of FP-CIT images in PD. Rather, earliest changes of SN-zSBR could be more sensitive biomarker for clinical probable DLB or MCI-LB. Considering higher burden of limbic/neocortical-predominant LRP and relatively lower burden of brainstem LRP in DLB^2,9,10^, this sequential pattern may suggest that SN-zSBR could be closely related with limbic/neocortical LRP as well as brainstem LRP.

Our third major finding was that regional zSBRs were associated with VH, severe parkinsonism, and cognitive dysfunction in patients with DLB spectrum. Previous studies showed negative findings on the association between striatal DAT uptake and core symptoms or cognition in DLB^33^. In this study, decreased nigrostriatal DAT uptake, except in the posterior caudate, was associated with the risk of VH. This is consistent with other studies that showed an inverse correlation between striatal DAT uptake with VH^34,35^. These findings are counterintuitive with the classic hypothesis of schizophrenia, which suggests that an overactive dopaminergic system, especially in the mesolimbic pathway, is associated with psychotic symptoms^36^. However, underlying mechanisms of hallucinations in schizophrenia are complex, and recent studies suggest that dysregulation of dopaminergic pathways, rather than simple hyper-dopamine status, is the main contributor^37^. Interestingly, one third of patients with schizophrenia show normal dopamine status^38^. Considering that dopaminergic medication does not decrease but can promote VH in PD^39,40^, the association between decreased zSBRs and VH may indicate that decreased zSBRs in DLB patients are not a direct cause of VH but reflect an increased risk of dopaminergic dysregulation that could originate from a dopaminergic imbalance between the associative/limbic striatum and the motor striatum.

While modest parkinsonism (UPDRS-III > 20) was not associated with nigrostriatal zSBRs, there was significant association between UPDRS-III motor score and nigrostriatal zSBRs. This is consistent with previous findings which showed correlation between severity of parkinsonism and striatal DAT uptake^41–43^. In terms of cognitive dysfunction, one study showed that caudate DAT uptake was associated with executive dysfunctions^42^, while other studies did not showed any correlation in MMSE^33,44^. Previous discrepancy could attribute to the methodological difference between detailed neuropsychological tests and general cognitive scores. In our study, only SN-zSBR was associated with K-MMSE score (Table 3), while analyses of detailed neuropsychological tests revealed association between nigrostriatal DAT uptake and cognitive dysfunction in multiple cognitive domains (Table 4). These results suggest that severe parkinsonism and dysfunction in the visuospatial and attention/executive domains in DLB spectrum, are closely related to nigrostriatal dopaminergic depletion in pathophysiological mechanisms.

Our fourth major finding was that lower SN-zSBR was associated with widespread GM atrophy in DLB spectrum. The GM regions associated with SN-zSBR overlapped with previously reported atrophy regions in patients with DLB, including the amygdala, inferior and lateral temporal, occipital, dorsolateral prefrontal, medial and lateral parietal, and basal frontal regions^45,46^. These results suggest that SN-zSBR could be biomarkers for disease progression in DLB spectrum. Moreover, as abnormalities in SN-zSBR preceded abnormalities in striatal zSBRs and had the best diagnostic accuracy among regional zSBRs to classify DLB or MCI-LB from HC, it could be suggested that SN-zSBR reflects early brain changes in the transition from healthy aging to DLB spectrum as well.

Our study had several limitations. First, the autopsy validation of the patients was not conducted, limiting the interpretation of abnormal FP-CIT images reflecting underlying α-synucleinopathy. Second, as we initially included patients with probable DLB or MCI-LB based on the core features of DLB without considering the results of FP-CIT PET and subsequently analyzed the subregional uptake pattern, we cannot exclude the possibility that patients with non-DLB, such as AD, were included in the study. Especially, longitudinal follow-up of the patients with MCI-LB is required to rule out the possibility of evolving the diagnosis to other diseases. Third, the HC group was not followed up for long periods; thus, the prodromal stage of underlying neurodegenerative disease, including α-synucleinopathy, could not completely be ruled out. Fourth, an age-related decrease in DAT activity^47–49^ could result in a decreased SN-zSBR. However, to avoid the possibility of non-specific aging-related decreases in DAT activity, we used age-adjusted z-scores of SBRs based on FP-CIT PET of HCs in all analyses. Fifth, our results are focused on DLB spectrum, which limits the application of our results to patients with PD. Although different patterns of striatal DAT uptake are reported between DLB and PD, nigral DAT uptake patterns in PD requires further research. Sixth, ventral tegmentum, the well-known neural correlates of VH, is also within the FWHM distance of the 6-mm smoothing kernel used in image analysis (Supplementary Fig. 4), which might contribute to the signals of SN-zSBR. Nonetheless, we conducted a comprehensive analysis of multiple features of DLB, including core clinical symptoms, cognitive functions, and GM density, in relation to nigrostriatal degeneration, especially focusing on DAT activity in the SN. Our data suggest that nigral dopaminergic uptake could be a sensitive biomarker of clinically probable DLB or MCI-LB. Additionally, it is possible that cascades of biomarker changes in DLB, from abnormal DAT uptake in the SN to cortical and limbic atrophy may lead to VH and cognitive dysfunction. This hypothesis requires further study with larger cohorts and longitudinal assessment of clinical features and FP-CIT PET.

## Methods

### Study participants

This study included 51 patients with probable DLB based on the fourth consortium criteria for DLB^2^. We also included 36 patients with prodromal stage DLB, which was defined as probable MCI-LB in accordance with established research criteria^5^. Diagnosis of probable DLB or probable MCI-LB was made when two or more core clinical features of DLB, including fluctuating cognition, recurrent visual hallucination, RBD, and parkinsonism, were present^2,5^. The distinction between DLB and MCI-LB was determined by disability in the activities of daily living. All patients visited the Dementia Outpatient Clinic at Severance Hospital, Yonsei University Health System, between January 2016 and October 2021. HCs (*n* = 40) were recruited as described in our previous study^50^. Clinical assessment, detailed neuropsychological tests, brain magnetic resonance imaging (MRI), and FP-CIT PET were conducted within 6 months. During the follow-up period of mean 3 years, there was no patient who exhibited clinical features of atypical parkinsonism, ophthalmoplegia, or ataxia. This study was approved by the Institutional Review Board of Severance Hospital, and written informed consent was obtained from all participants.

### Clinical assessment

Clinical symptoms of DLB, including RBD, VH, and CF, were evaluated using structured questionnaires administered by caregivers (Supplementary Table 5). Motor severity was measured using the modified UPDRS-III score. The presence of modest parkinsonism was defined as a UPDRS-III score of ≥20.

### Neuropsychological evaluation

All participants underwent a standardized neuropsychological battery called the Seoul Neuropsychological Screening Battery^51^ that included the following scorable tests: digit span (forward and backward), K-BNT, SVLT (immediate recall, 20 min delayed recall, and recognition tests), RCFT (copying, immediate recall, 20 min delayed recall, and recognition tests), clock drawing test, set-shifting ability (go-no-go), phonemic and semantic COWAT, Stroop word reading, Stroop color reading, digit symbol coding, and Korean-trail making test^52^. Age- and education-specific norms were based on 1067 healthy and cognitively healthy community-dwelling individuals. General cognitive status was measured using the K-MMSE and the CDR-SOB.

### Acquisition and image processing of MRI and PET

The participants were scanned using a Philips 3.0 T MR scanner (Philips Achieva; Philips Medical Systems, Best, The Netherlands) with a SENSE head coil (SENSE factor = 2). T1-weighted MRI data were obtained using a three dimensional (3D) T1-weighted turbo field echo (T1-TFE) sequence with the following parameters: axial acquisition matrix, 224 × 224; reconstructed matrix, 256 × 256 with 170 slices; voxel size, 0.859 × 0.859 × 1 mm^3^; field of view, 220 mm; echo time, 4.6 ms; repetition time, 9.8 ms; and flip angle, 8°.

FP-CIT PET was acquired using Discovery 600 (General Electric Healthcare, Milwaukee, MI, USA). After 90 min of administration of approximately 185 MBq (5 mCi) FP-CIT^53^, PET images were acquired for 15 min with a 256 × 256 matrix and reconstructed with an ordered-subsets expectation-maximization algorithm in an iso-0.98 mm voxel size.

### Image processing

The FMRIB Software Library (FSL, http://www.fmrib.ox.ac.uk/fsl) was used for T1-weighted image (T1w) processing. T1w MRIs were corrected for intensity non-uniformities using the N3 algorithm. The images were skull-stripped and linearly registered into the Alzheimer’s Disease Neuroimaging Initiative (ADNI)–Montreal Neurological Institute (MNI) atlas, a T1w template for older adults^54^. Brain tissues were classified as white matter, GM, or cerebrospinal fluid (CSF) based on the hidden Markov random field model and the associated expectation-maximization algorithm^55^. The GM probability maps were nonlinearly transformed into the MNI–ADNI template. The resulting images were flipped and averaged to create a study-specific symmetrical template. Native GM probability maps were spatially re-registered to the customized template in the next iteration and modulated to maintain the total GM amount constant, regardless of local expansion or contraction due to image normalization. To generate a study-specific GM mask, we averaged all the individual GM probability maps and binarized above 30% of the map. Striatal subregions were segmented using the FSL FIRST algorithm^56^, and the SN region was defined based on the automated anatomical labeling (AAL) atlas (version 3)^57^ and high deformable registration (SyN) algorithm implemented in the Advanced Normalization Tools^58^. The occipital white matter mask was created by combining individual tissue classification results with the MNI structural atlas included in the FSL package. Intracranial volumes (ICVs) were calculated by summing the GM, white matter, and CSF classes within the skull mask in the individual native space.

FP-CIT PET scans were co-registered to individual T1w, and standardized uptake value ratio maps of the FP-CIT PET images were generated using the occipital white matter uptake as a reference^59^. We extracted the median FP-CIT uptake from the following five ROIs: SN, bilateral AC, PC, AP, and PP. The anterior and posterior regions of the caudate and putamen were subdivided using the k-means clustering algorithm based on the voxel coordinates. We eroded one voxel from each striatal ROI during extraction to minimize the partial volume effect.

### Quality assurance for image processing

All MRI and PET images and processing results from the automated pipelines were visually inspected by three researchers (YGL, SJ, and BSY) blinded to the participant information for quality assurance.

### Statistical analysis

Statistical analyses of the demographic and clinical data were performed using R statistical software (version 4.1.0). Imaging analyses with voxel-wise statistics were performed using the SurfStat toolbox (http://www.math.mcgill.ca/keith/surfstat/). The chi-square test or analysis of variance was used to compare the demographic variables.

SBRs of the HC group showed an age-dependent decrement (*p* < 0.001; Supplementary Fig. 5), although they were not associated with education (data are not shown). To compute the zSBR in each participant in the DLB and MCI-LB group, we generated GLMs for SBRs using age at FP-CIT PET as a predictor in the HC group. Subsequently, predicted mean and residual standard errors based on the GLMs in the HC group were used to compute the zSBR of each patient with DLB or MCI-LB.

To evaluate the performance of nigrostriatal FP-CIT uptake in the classification of DLB or MCI-LB from HCs, univariate logistic regression was performed for the combined group consisting of DLB or MCI-LB and HC. Also, ROC curve analyses were performed using zSBRs as predictors, and the AUC was calculated. To determine whether the mean zSBRs of the bilateral nigrostriatal ROIs reduced the diagnostic accuracy, the performance of SBRs for the diagnostic accuracy for DLB was further evaluated using the zSBRs of the more affected side as predictors.

To assess the sequential order of abnormal DAT uptake, we conducted conditional probability analysis using zSBRs separately in DLB or MCI-LB group. We defined abnormal DAT in a subregion Y as having an zSBR less than −1.5. We subsequently computed the probability of abnormal DAT uptake of the subregion Y (*Y*+) provided normal DAT uptake of the subregion X $\left(X-\right)$, denoted as ${\rm{P}}\left(Y+|X-\right)=P(Y+\cap X-)/P\left(X-\right)$. With the null hypothesis ${\rm{P}}\left(Y+|X-\right)={\rm{P}}\left(X+|Y-\right)$, the significance of the conditional probability was tested using McNemar’s test.

To evaluate the independent effect of zSBRs on the clinical symptoms of DLB and MCI-LB group, we performed logistic regression analyses for the presence of RBD, CF, VH, or parkinsonism using zSBRs as predictors in the DLB and MCI-LB group after controlling for age, sex, and education. We also performed GLMs for UPDRS-III and neuropsychological test scores using the same covariates. The false discovery rate (FDR) method was used to correct for multiple comparisons across the five zSBRs. In the sensitivity analysis, we further considered the medication history of antipsychotics in the logistic regression analysis for the presence of VH.

To evaluate the association between zSBRs and brain atrophy, we performed GLMs for voxel-wise GM density, using age, sex, education, and ICV as covariates. In voxel-based analyses, we employed the random field theory (RFT) cluster correction method to account for spatial relationships among adjacent voxels. This was achieved using the SurfStat toolbox, which implements RFT-based cluster correction. The cluster-forming threshold was set to *p* < 0.001, and the cluster size threshold was determined by permutation testing. We displayed voxel-wise statistical outcomes, including effect sizes (r), within statistically significant areas (corrected *p* < 0.05, family-wise error rate) on the stereotaxic space in the neurological convention.

### Reporting summary

Further information on research design is available in the Nature Portfolio Reporting Summary linked to this article.

## Supplementary information

SUPPLEMENTAL MATERIAL

Reporting summary

## Data Availability

The data supporting the findings of this study are available from the corresponding author upon reasonable request by any qualified investigator.
