# Supplementary material for: Substantia nigral dopamine transporter uptake in dementia with Lewy bodies
Source: NPJ Parkinsons Dis. 2023 Jun 9;9:88. doi: 10.1038/s41531-023-00534-9 (PMC10256694; doi:10.1038/s41531-023-00534-9)
Supplement: Supplementary file 1 — SUPPLEMENTAL MATERIAL [file 41531_2023_534_MOESM1_ESM.pdf]

Supplementary Table 1. Logistic regression analyses for the effects of nigrostriatal zSBRs on the risk of DLB or MCI-LB

Outcome	Predictors	OR (95% CI)	*P*	AIC
DLB versus HCs	AP-zSBR	0.51 (0.35 – 0.70)	< **0.001**	108.8
	PP-zSBR	0.58 (0.42 – 0.77)	< **0.001**	112.8
	AC-zSBR	0.47 (0.31 – 0.66)	< **0.001**	105.3
	PC-zSBR	0.44 (0.27 – 0.67)	< **0.001**	111.7
	SN-zSBR	0.20 (0.10 – 0.35)	< **0.001**	72.9
MCI-LB versus HCs	AP-zSBR	0.54 (0.33 – 0.81)	**0.006**	99.7
	PP-zSBR	0.62 (0.40 – 0.89)	**0.018**	102.4
	AC-zSBR	0.46 (0.27 – 0.71)	**0.001**	94.8
	PC-zSBR	0.48 (0.28 – 0.76)	**0.004**	98.8
	SN-zSBR	0.23 (0.11 – 0.43)	< **0.001**	76.8

Abbreviations: AC, anterior caudate; AIC, Akaike's Information Criterion; AP, anterior putamen; DLB, dementia with Lewy bodies; HC, healthy control; MCI-LB, mild cognitive impairment with Lewy body; PC, posterior caudate; PP, posterior putamen; zSBR, age-adjusted z-score of specific binding ratio; SN, substantia nigra.

Data are results of univariable logistic regression analyses for the diagnosis of DLB or MCI-LB versus HCs using nigrostriatal zSBRs as predictors. *P* values in bold is significant after multiple comparisons corrections across five predictors using the false discovery rate (FDR) method.

Supplementary Table 2. Receiver operating characteristic analyses for the diagnosis of DLB or MCI-LB

Outcome	Predictors	Sensitivity	Specificity	PPV	NPV	AUC (95% CI)	Cutoff value
DLB versus HCs	AP-zSBR	56.9	97.5	60.7	63.9	0.74 (0.64 – 0.85)	-1.40
	PP-zSBR	49.0	97.5	96.2	60.0	0.72 (0.61 – 0.82)	-1.83
	AC-zSBR	66.7	87.5	87.2	67.3	0.77 (0.68 – 0.87)	-0.91
	PC-zSBR	72.5	67.5	74.0	65.9	0.74 (0.64 – 0.84)	-0.42
	SN-zSBR	82.4	97.5	97.7	81.2	0.91 (0.85 – 0.98)	-1.08
MCI-LB versus HCs	AP-zSBR	47.2	90.0	81.0	65.5	0.72 (0.60 – 0.84)	-0.97
	PP-zSBR	55.6	80.0	71.4	66.7	0.65 (0.53 – 0.78)	-0.81
	AC-zSBR	58.3	90.0	84.0	70.6	0.76 (0.65 – 0.87)	-0.97
	PC-zSBR	52.8	80.0	70.4	65.3	0.71 (0.69 – 0.82)	-0.97
	SN-zSBR	75.0	90.0	87.1	80.0	0.87 (0.79 – 0.96)	-0.72

Abbreviations: AC, anterior caudate; AP, anterior putamen; AUC, area under curve; CI, confidence interval; DLB, dementia with Lewy bodies; HC, healthy control; MCI-LB, mild cognitive impairment with Lewy body; NPV, negative predicted value; PC, posterior caudate; PP, posterior putamen; PPV, positive predicted value; SN, substantia nigra; zSBR, age-adjusted z-score of specific binding ratio.

Data are results of receiver operating characteristic analyses for the diagnosis of DLB or MCI-LB versus HCs using nigrostriatal zSBRs as predictors.

Supplementary Table 3. Receiver operating characteristic analyses for the diagnosis of DLB or MCI-LB using combined striatal zSBR

Outcome	Predictors	Sensitivity	Specificity	PPV	NPV	AUC (95% CI)	Cutoff value
DLB versus HCs	Striatal-zSBR	58.8	92.5	90.9	63.8	0.78 (0.68 – 0.87)	-1.25
MCI-LB versus HCs	Striatal-zSBR	72.2	70.0	68.4	73.7	0.74 (0.63 – 0.86)	-0.36

Abbreviations: AUC, area under curve; CI, confidence interval; DLB, dementia with Lewy bodies; HC, healthy control; MCI-LB, mild cognitive impairment with Lewy body; NPV, negative predicted value; PPV, positive predicted value; zSBR, age-adjusted z-score of specific binding ratio.

Data are results of receiver operating characteristic analyses for the diagnosis of DLB or MCI-LB versus HCs using nigrostriatal zSBRs as predictors.

Supplementary Table 4. Sensitivity analysis for the effects of nigrostriatal FP-CIT zSBRs on visual hallucination in the overall patient group

	Visual hallucination	
	OR (95% CI)	*P*
AP-zSBR	0.59 (0.40 – 0.83)	**0.004**
PP-zSBR	0.67 (0.49 – 0.89)	**0.009**
AC-zSBR	0.62 (0.42 – 0.88)	**0.011**
PC-zSBR	0.64 (0.38 – 1.03)	0.074
SN-zSBR	0.58 (0.37 – 0.87)	**0.012**

Abbreviations: AC, anterior caudate; AP, anterior putamen; CI, confidence interval; DLB, dementia with Lewy bodies; MCI-LB, mild cognitive impairment with Lewy body; OR, odds ratio; PC, posterior caudate; PP, posterior putamen; SN, substantia nigra; zSBR, age-adjusted z-score of specific binding ratio.

Data are results of logistic regression analyses for the presence of visual hallucinations using nigrostriatal zSBRs as predictors in patients of DLB and MCI-LB. Covariates included age, sex, education, and the medication history of antipsychotics. *P* values in bold are significant after multiple comparisons corrections across five predictors using the false discovery rate (FDR) method.

Supplementary Table 5. Structured questionnaire for the clinical features of dementia with Lewy bodies

Clinical symptoms	Main question	Selective answer
REM sleep behavior disorder	Muttering or making noise while sleeping. Hitting the person next to you. Putting the contents of your dreams into action while you sleep.	Yes
		No
Visual hallucination	Sometimes I see things that don't really exist.	None
		Right after waking up, there are times when I mistake or confuse things for people.
		When your consciousness is clear, there are times when I mistake or confuse things for people.
		Even when conscious, there are times when I see small animals/bugs that are clearly colored.
Cognitive fluctuation	There is a change in cognitive function to the extent that a person who does not know the patient well may feel that he/she is a completely different person when he/she sees the patient several times (Sometimes it's normal or good, other times it gets really bad.)	None
		It's to the extent that it looks a bit dazed at times.
		Talking nonsense or being confused, making conversation and daily life difficult.

Supplementary Figure 1. Comparisons of SBRs between MCI-LB and DLB.

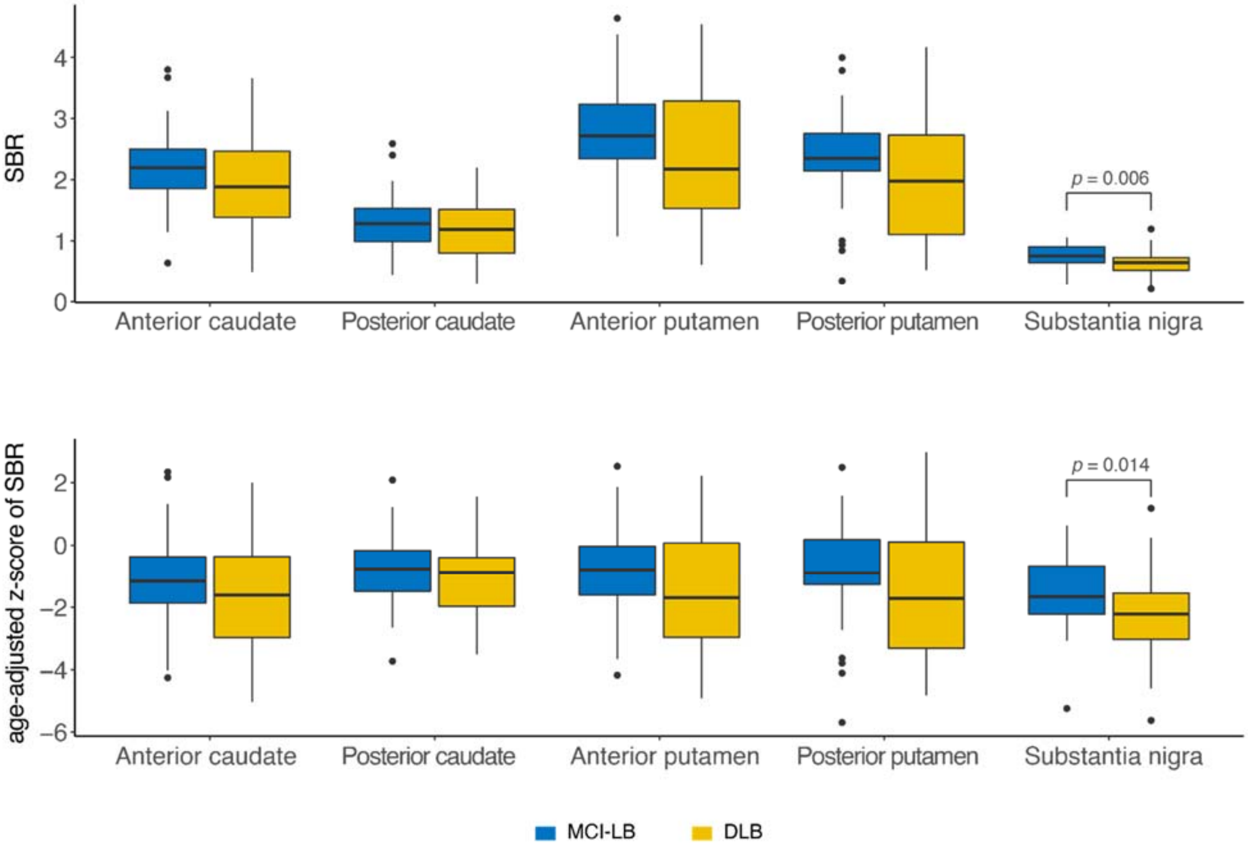

Comparisons of SBRs and age-adjusted z-score of SBRs between patients with MCI-LB and DLB. Significant *P* values from t-test was denoted. DLB, dementia with Lewy bodies; MCI-LB, mild cognitive impairment with Lewy body; SBR, specific binding ratio. The central lines within boxes correspond to the median; box limits correspond to upper and lower quartiles; whiskers correspond to 1.5 × interquartile range; spots correspond to outliers.

Supplementary Figure 2. ROC curve analyses for the diagnostic accuracy of DLB and MCI-LB using nigrostriatal zSBRs of the more affected side.

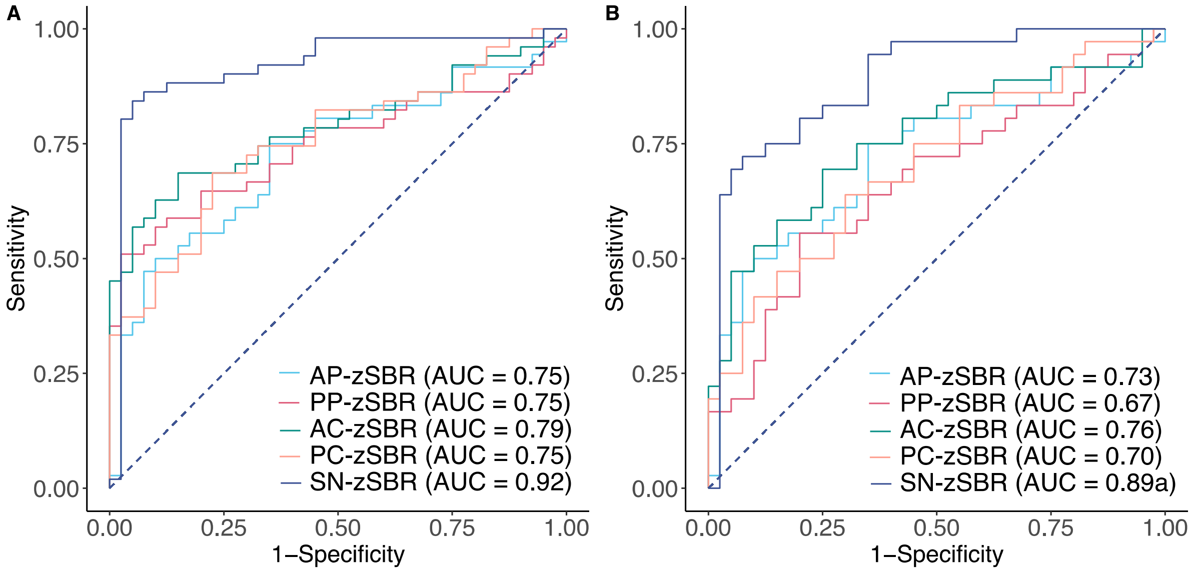

The results of ROC analyses for the diagnostic accuracy of DLB (A) and MCI-LB (B) versus HCs using nigrostriatal zSBRs of the more affected side as predictors. AC, anterior caudate; AP, anterior putamen; AUC, area under the curve; DLB, dementia with Lewy bodies; HC, healthy control; MCI-LB, mild cognitive impairment with Lewy body; PC, posterior caudate; PP, posterior putamen; ROC, receiver operating characteristic; SN, substantia nigra; zSBR, age-adjusted z-score of specific binding ratio

Supplementary Figure 3. Association of nigrostriatal zSBRs with parkinsonism and general cognition in the overall patient group.

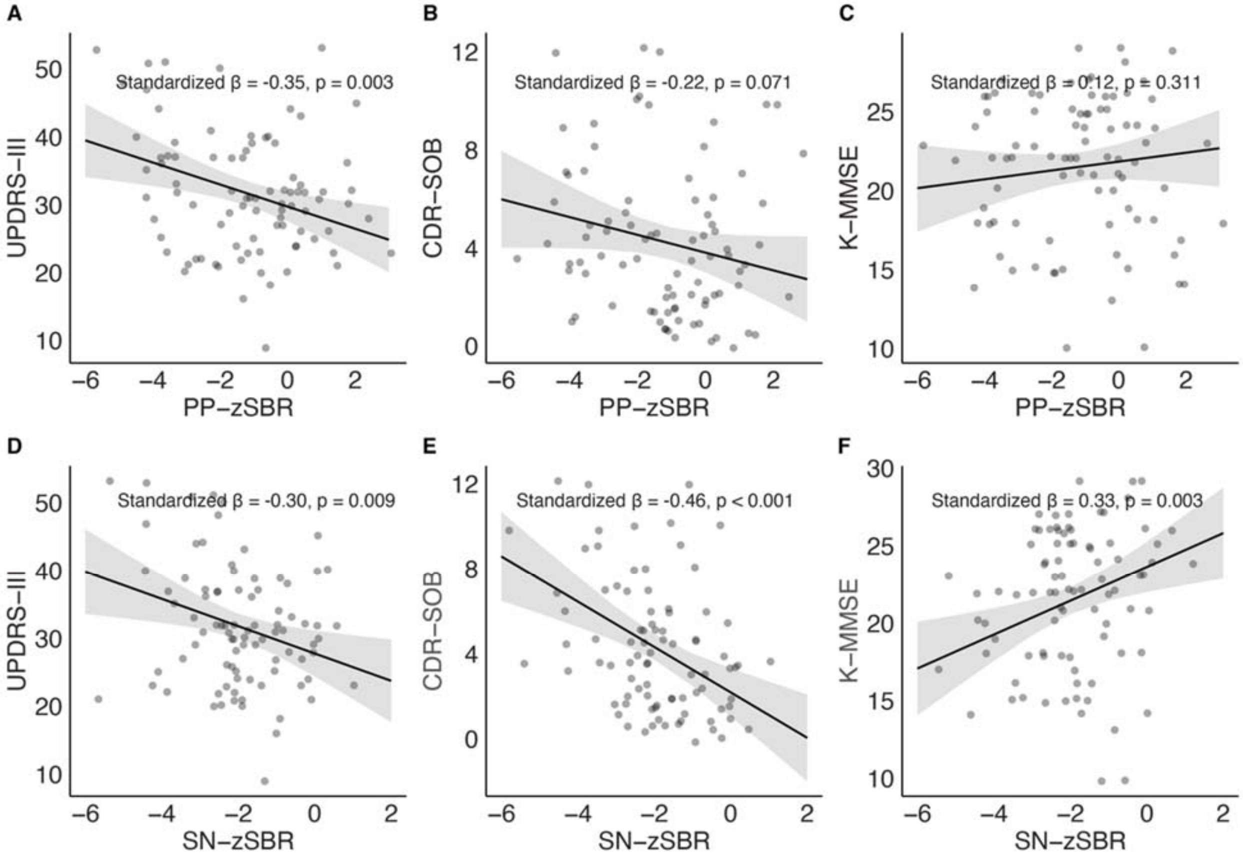

Data are the results of general linear models for UPDRS-III (A and D), CDR-SOB (B and E), and K-MMSE (C and F) in patients of DLB and MCI-LB using PP-zSBR (A, B, and C) or SN-zSBR (D, E, and F) as predictors. Covariates included age, sex, and education. Each dot represents individuals and predicted regression lines (black line) with confidence intervals (gray shade) are displayed. CDR-SOB, Clinical Dementia Rating-sum of boxes; DLB, dementia with Lewy bodies; K-MMSE, Korean version of Mini-Mental State Examination; MCI-LB, mild cognitive impairment with Lewy body; PP, posterior putamen; SN, substantia nigra; UPDRS-III, Unified Parkinson's Disease Rating Scale Part III; zSBR, age-adjusted z-score of specific binding ratio.

Supplementary Figure 4. Regions of interest for the ventral tegmentum, raphe nucleus, and substantia nigra.

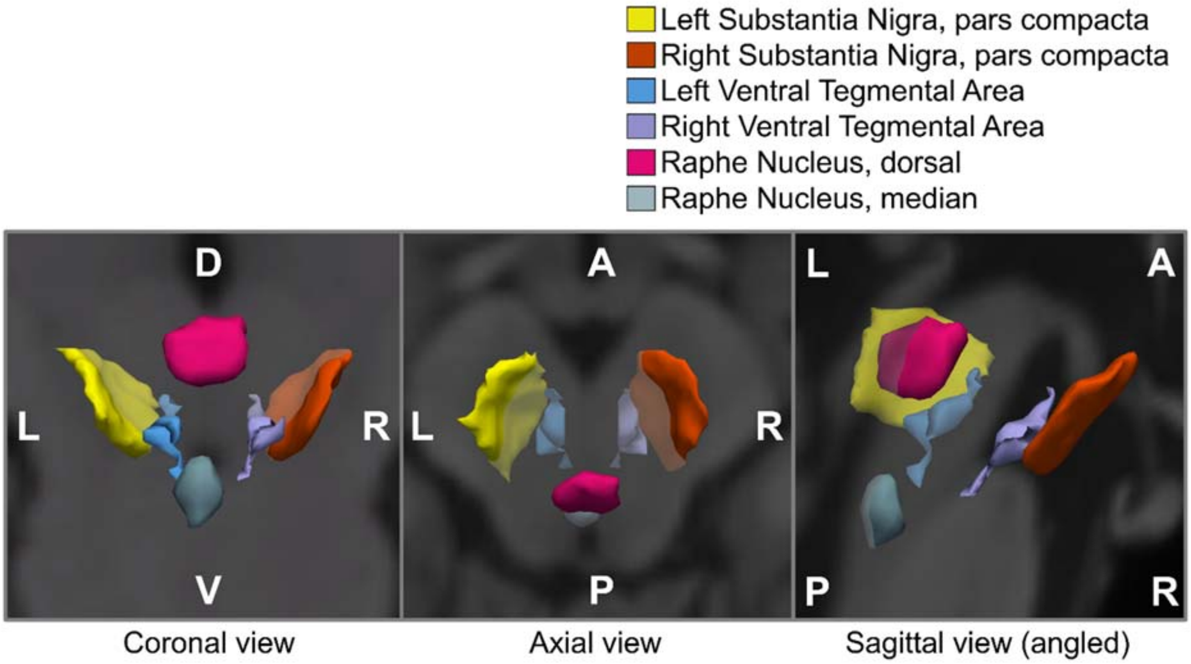

Bilateral ventral tegmentum, raphe nucleus, and substantia nigra are displayed on coronal, axial, and sagittal slices of MR images.

Supplementary Figure 5. Age-dependent changes of SBRs in the healthy control group.

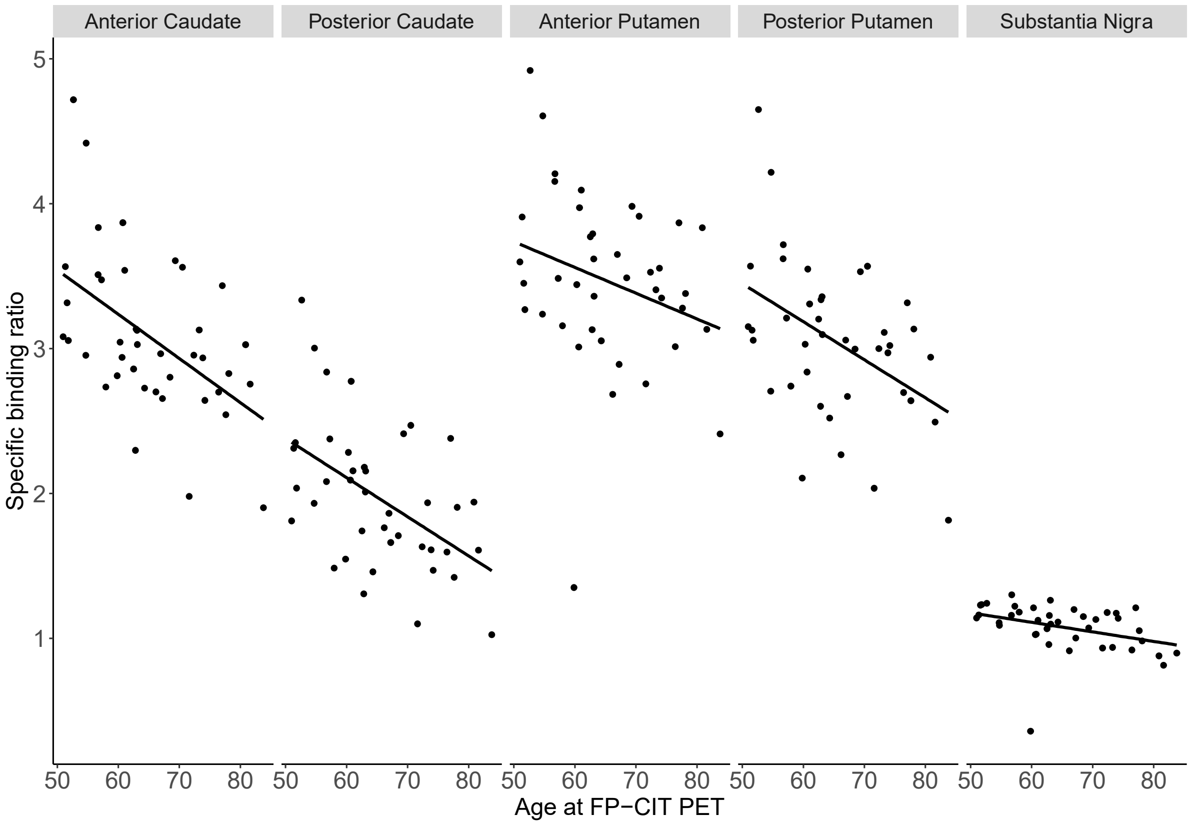

Association between age at FP-CIT PET and nigrostriatal SBRs in healthy control group (n = 40). Each dot represents individuals and predicted regression lines (black solid line) with confidence intervals (gray shade) are displayed. SBR, specific binding ratio.
